# A survey exploring self-reported indoor and outdoor footwear habits, foot problems and fall status in people with stroke and Parkinson’s

**DOI:** 10.1186/s13047-016-0170-5

**Published:** 2016-09-22

**Authors:** Catherine Bowen, Ann Ashburn, Mark Cole, Margaret Donovan-Hall, Malcolm Burnett, Judy Robison, Louis Mamode, Ruth Pickering, Dan Bader, Dorit Kunkel

**Affiliations:** 1 Faculty of Health Sciences, University of Southampton, Southampton, UK; 2 Faculty of Medicine, University of Southampton, Southampton, UK

**Keywords:** Shoes, Footwear, Stroke, Parkinson’s, Podiatry

## Abstract

**Background:**

Ill-fitting shoes have been implicated as a risk factor for falls but research to date has focused on people with arthritis, diabetes and the general older population; little is known about people with neurological conditions. This survey for people with stroke and Parkinson’s explored people’s choice of indoor and outdoor footwear, foot problems and fall history.

**Methods:**

Following ethical approval, 1000 anonymous postal questionnaires were distributed to health professionals, leads of Parkinson’s UK groups and stroke clubs in the wider Southampton area, UK. These collaborators handed out survey packs to people with a confirmed diagnosis of stroke or Parkinson’s.

**Results:**

Three hundred and sixty three completed surveys were returned (218 from people with Parkinson’s and 145 from people with stroke). Most respondents wore slippers indoors and walking shoes outdoors and considered comfort and fit the most important factors when buying footwear. Foot problems were reported by 43 % (95 % confidence intervals 36 to 52 %; stroke) and 53 % (95 % confidence interval 46 to 59 %; Parkinson’s) of respondents; over 50 % had never accessed foot care support. Fifty percent of all respondents reported falls. In comparison to non-fallers, a greater proportion of fallers reported foot problems (57 %), with greater proportions reporting problems impacting on balance and influencing choice of footwear (*p* < 0.01) in comparison to non-fallers in each case. Forty-seven percent of fallers with foot problems had not accessed foot care support.

**Conclusions:**

Many people with stroke and Parkinson’s wear slippers indoors. A high percentage of these individuals reported both foot problems and falls impacting on footwear habits and choice of footwear; however many did not receive foot care support. These findings highlight that further exploration of footwear and foot problems in these populations is warranted to provide evidence based advice on safe and appropriate footwear to support rehabilitation and fall prevention.

**Electronic supplementary material:**

The online version of this article (doi:10.1186/s13047-016-0170-5) contains supplementary material, which is available to authorized users.

## Background

There is convincing evidence that foot problems and inappropriate footwear affect balance [1] and increase the risk of falls in the older population [2–4]. In older people, shoes have been recognised as a modifiable risk factor in falls prevention [5]. Evidence from the general population suggests that appropriate footwear can enhance well being, allow maintenance of independence, mobility and freedom from pain [5–9]. Although the influence of footwear on balance is complex, some characteristics have been linked to positive health benefits and others to foot pathology [6, 7]. For example, appropriate footwear can enhance foot health including protection, support and facilitate propulsion [8]. Appropriate footwear can be defined as well fitting, fit for purporse supportive shoes that allow normal foot function [10]. Features associated with healthy footwear include a stable heel approximately 25 mm high, that have means of fastening, adequate width and depth, outsole grip that meets industry standard, soft flexible uppers and an inshoe climate that promotes a healthy environment within the shoe [10].

In contrast, specific styles of shoes such as slippers, high heeled court shoes and shoes with limited or absent fixation are notably associated with sub-optimal characteristics [9]. Factors such as excessive heel height, reduced friction on the soles of footwear, walking barefoot, wearing socks or footwear with a flimsy sole have all been linked to instability and increased fall risk in older people [11, 12]. Other characteristics such as heel collar height, heel and midsole geometry, type of fastening and sole hardness have also been shown to negatively influence balance performance [13, 14]. Indeed, walking barefoot and wearing stockings or socks without shoes was associated with a ten-fold increased risk of falling [15]. Wearing slippers or medium to high heeled shoes and shoes with a narrow heel significantly increased the likelihood of a fracture [16]. Much of the work on footwear and falls prevention has concentrated on therapeutic or off the shelf footwear intended for outside usage with little attention on indoor footwear [4, 12]. As falls frequently occur within the home setting [3] investigating individual’s choice of indoor footwear and safe mobility in the home may lead to a greater awareness of modifiable factors.

People with Parkinsons (PwP) and people with stroke (PwS) are at higher risk of falls than people among the general population. In a 12-month period two thirds of PwP [17] and three-quarters of PwS [18] living in the community will have fallen at least once. Most falls among these groups of people take place in the home where they spend most of their time [19]. The consequences of falls include injuries, loss of independence and social isolation often resulting in poor quality of life for the individual and high health service costs [20–22].

Little is known about footwear and foot problems for PwS or PwP. Rehabilitation for PwS and PwP currently focuses mainly on gait characteristics and gross motor performance of the lower limb but feet are often not included in routine assessment and treatment [20–22]. Post stroke, foot and toe deformities, altered sensation and proprioception have been observed [23–27] but their impact on balance is still unclear and no information exists as to whether these problems can be minimised with appropriate footwear. There is a lack of specific information about what PwS and PwP wear indoors and outdoors and no condition specific information about footwear characteristics that may enhance balance performance or which factors might be linked with instability and increased risk of falls in these conditions.

Evidence from a qualitative study among community dwelling people with stroke highlighted the contribution of pain, weakness and altered sensation to problems with community ambulation, self-esteem and perceptions of physical appearance [20]. In the current study we set out to explore what PwP and PwS wear on their feet in different settings (home indoors and outdoors) and to gather information about self-reported foot problems, fall status and factors that influence choice of footwear and buying decisions.

## Method

### Study design

A quantitative postal survey design was used involving open and closed questions.

### Study sample

Participants were identified from hospital clinics and consultant lists, out-patient services and clinics, and local support groups (Parkinson’s UK and Stroke Clubs) in the wider Southampton area.

### Procedure

Two anonymous questionnaires, one for each condition group (PwP, PwS), were formulated by the research team with PPI involvement. Each questionnaire comprised 22 questions. They included closed (yes/no and multiple choice) questions, ranking questions and open questions for free text responses. Information was requested on demographic data, time since diagnosis, ability to walk inside and outside, walking aid use, fall history relating to falls experienced in the previous 12 months (and for PwP a question about freezing of gait). Questions related to preferred footwear in the home and outdoors used pictorial selection and were adapted from previous investigators' work [28]. Participants were asked whether they had experienced foot problems and given free space to describe them. They were also asked if they had received foot care support from health care professionals and if their footwear habits had changed since the onset of their condition. In relation to purchasing new shoes, participants were asked to rank the factors that most influence their shoe purchasing decisions. The questionnaires were piloted with 10 participants before 1000 questionnaires (500 for PwP and 500 for PwS) were distributed to health professionals, leads of Parkinson’s UK groups and stroke clubs within the wider Southampton area (May 2014-May 2015). These collaborators were asked to distribute the survey packs to people with a confirmed diagnosis of either stroke or Parkinson’s.

### Governance

Each survey pack contained an information sheet, the anonymous survey and a Freepost envelope. As this survey is part of the larger, multi-study SHOES research project (NIHR RfPB: PB-PG-0212-27001) the information sheet clearly described each phase of the study. In the survey stage consent was implied from participating in the survey by returning the completed questionnaire. Full ethical approval was granted through the UK IRAS (Integrated Research Application System) (LREC: 14/SW/0078); Research Governance was approved by University Hospitals Southampton NHS Foundation Trust (R&D: RHM MED 1169).

### Statistical analysis

Demographic and clinical characteristics were described using summary statistics. The footwear specific characteristics and foot problems in each condition group were presented as frequencies of occurrence and graphically as bar charts. Free text responses were analysed quantitatively by counting the number of similar responses and then representing the textual content numerically. The percentage of participants with foot problems was reported. Pearson chi-squared analyses and independent sample t-tests (Mann-Whitney U tests were performed when assumptions of normality or homogeneity were not met) were used to explore differences between PwP and PwS, between those with and without foot problems, and between fallers and non-fallers. Analyses were conducted using Statistical Package for the Social Sciences version 22.0 software (IBM SPSS, Chicago IL) and 95 % confidence intervals from programme CIA. *P* values less than 0.05 were deemed to indicate statistical significance.

## Results

A total of 363 surveys were completed by 218 PwP and 145 PwS, and returned to the research centre (see Table 1 for participant characteristics). Whilst more PwP than PwS responded to the questionnaire the condition groups were similar in age, living status and fall status.

**Table 1:** Characteristics of the participants in each condition group

		PwP (*n* = 218)	PwS (*n* = 145)
Age (years)	mean (min to max)	72.52 (50 to 93)	72.35 (40 to 93)
Gender	Male	124 (57 %)	72 (50 %)
	Female	93 (43)	73 (50 %)
Time (months) since stroke onset/ PD diagnosis.	mean (min to max)	69.25 (1–300)	45.03 (1–386)
Living status	On own	40 (18 %)	41 (28 %)
	With partner	151 (69 %)	85 (59 %)
	With family	20 (9 %)	16 (11 %)
	Residential home	2 (1 %)	1 (1 %)
	Warden controlled apartment	2 (1 %)	2 (1 %)
	Live-in carer	3 (2 %)	
Mobility	Nonfunctional,	2 (1 %)	10 (7 %)
	Dependent, Level II	17 (8 %)	8 (6 %)
	Dependent Level I	21 (10 %)	17 (12 %)
	Dependent Supervision	10 (5 %)	10 (7 %)
	Independent, Level surfaces	54 (25 %)	46 (32 %)
	Independent, Level and non-Level surfaces	108 (50 %)	49 (34 %)
Reported falls per year	Non-faller	108 (50 %)	75 (52 %)
	Faller	110 (50 %)	70 (48 %)
	*One time faller*	*42 (19 %)*	*35 (24 %)*
	*Repeat faller*	*68 (31 %)*	*35 (24 %)*
Walking aids		109 (50 %)	99 (68 %)

Figures are number(%) unless stated otherwise

### Footwear

**Fig. 1:**
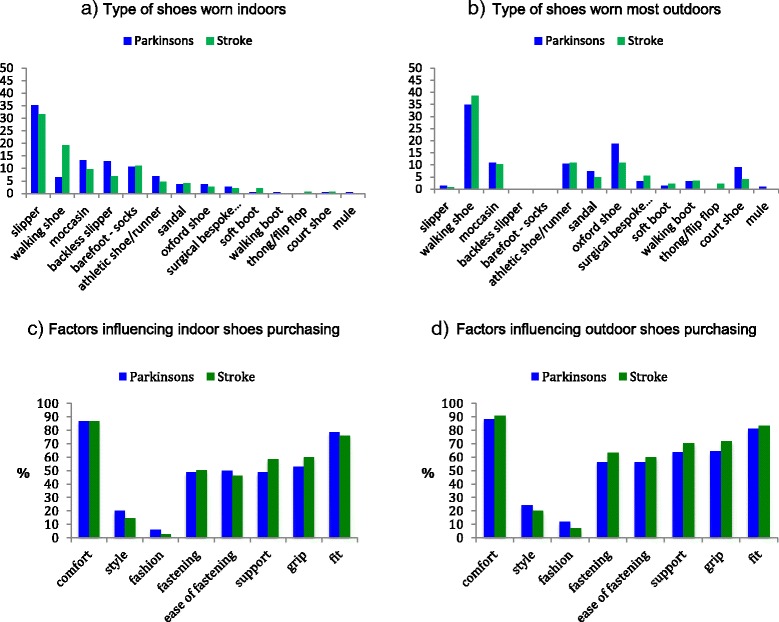
Footwear habits of people with Parkinsons and people with stroke. **a**) Type of shoes worn indoors; **b**) Type of shoes worn outdoors; **c**) factors influencing indoor shoes purchasing; **d**) Factors influencing outdoor shoes purchasing

Over 40 % of respondents reported that they had received foot care help, and that their footwear habits had changed since the onset/diagnosis of their condition (see Table 2). In the free text responses a number of PwS and PwP reported that they stopped wearing higher heeled shoes, now favouring more sturdy, supportive and comfortable shoes with slip on or velcro fastening shoes (due to problems in doing up laces). PwS reported they made changes in response to problems with swelling, pain, lack of sensation, weakness or having to purchase two different shoes sizes to accommodate ankle foot orthosis. PwP reported a new focus on wider fit, lighter, non slip, easy to put on and off shoes with better fastening and indicated regret that choices were less governed by fashion in favour of safety. The type of shoes most often reported as being worn indoors were slippers (PwP: 35 %; PwS: 32 %), see Fig. 1a. Walking shoes were most frequently reported as being worn outdoors (PwP:35 %; PwS: 39 %), see Fig. 1b. The majority believed that their current shoes were right for them; both for indoor (65 % PwP and 70 % PwS) and outdoor shoes (76 % of PwP and 80 % PwS); data not shown in table. PwS and PwP who described problems with their current shoes mentioned problems with doing up laces, discomfort and pain, tightness, wearing down one side of the shoe, heaviness or problems in putting shoes on and off. PwP also mentioned problems with day to day variability which led to problems finding shoes that were right for them. The main stroke specific issues were having to accommodate orthosis or cope with swelling as this often led to shoes being too tight, too wide or too big as a result. Visual inspection of the responses suggested no substantial differences in footwear choices between those who did and did not receive foot care help. For example of the 196 respondents who reported that they mostly wore slippers or walked barefoot indoors, 89 (45 %) received foot care help while 107 (55 %) did not.

**Table 2:** Survey answers for people with Parkinson’s and people with stroke relating to foot problems and footwear

		PwP (*n* = 218)	PwS (*n* = 145)	*P* value
Do you receive foot care help from a health professional? (% yes)		101 (46 %)	62 (43 %)	0.321
Have your footwear habits changed since diagnosis? (% yes)		96 (44 %)	68 (47 %)	0.531
Foot problems?	any problem	115 (53 %)	63 (43 %)	0.082
	influencing balance	56 (26 %)	44 (30 %)	0.282
	influencing shoes worn	70 (32 %)	39 (27 %)	0.289
Would like more choice when buying footwear?		86 (39 %)	66 (46 %)	0.027
Would like more advice when buying footwear?		72 (33 %)	62 (43 %)	0.010
Factors influencing indoor footwear purchase? (% ranking factor ‘important’)	Comfort	189 (87 %)	126 (87 %)	0.401
	Style	44 (20 %)	21 (14 %)	0.038
	Fashion	13 (6 %)	4 (3 %)	0.133
	Secure fastening	106 (49 %)	73 (50 %)	0.609
	Ease of fastening	109 (50 %)	67 (46 %)	0.073
	Support	106 (49 %)	85 (59 %)	0.888
	Grip	115 (53 %)	87 (60 %)	0.882
	Fit	171 (78 %)	110 (76 %)	0.046
Factors influencing outdoor footwear purchase? (% ranking factor ‘important’)	Comfort	192 (88 %)	132 (91 %)	0.949
	Style	53 (24 %)	29 (20 %)	0.261
	Fashion	26 (12 %)	10 (7 %)	0.141
	Secure fastening	123 (56 %)	92 (63 %)	0.793
	Ease of fastening	123 (56 %)	87 (60 %)	0.945
	Support	139 (64 %)	102 (70 %)	0.998
	Grip	140 (64 %)	104 (72 %)	0.875
	Fit	177 (81 %)	121 (83 %)	0.904

Pearson chi-square test comparing yes/no responses or important/not important responses

### Factors affecting decisions on purchasing shoes

The most important factors that impacted on decisions for purchasing indoor and outdoor shoes were reported to be comfort and fit; followed by fastening, grip and support (see Table 2 and Fig. 1c and d). The only differences in factors that impacted on purchases was that a greater proportion of PwP rated style and fit of indoor shoes as important in comparison to PwS, and a higher proportion of PwS would like more choice and advice when buying shoes.

### Foot problems

Foot problems were reported by half PwP and just under half of PwS (see Table 2) and approximately a third of respondents reported that foot problems influenced balance and the types of shoes they wear. Just under half of all respondents reported receiving foot-care from a health professional, however not all participants who reported foot problems received foot care help from a health professional. Further exploration of this data showed that 20 % of participants who reported foot problems did not receive help (data not shown). There was a significant difference in time since onset between those who did and did not receive support from health professionals. The average time since onset for those receiving help was 77 months in comparison to 46 months for those not receiving help (data not shown). Similarly, those who reported foot problems had been living with their condition for longer (on average 70 months) in comparison to those without foot problems, (*p* < 0.01, data not shown). Swelling of the feet/ankles, corns and callus were the most frequently reported foot problems for PwP; weakness, limited movement in the feet/ankles, loss of sensation and pain were the most frequently reported foot problems for PwS (see Table 3).

**Table 3:** Self-reported foot problems ranked by order of most reported

	PwPD	PwS
1.	Swelling in feet / ankles	Weakness and limited movement in feet/ankles
2.	Corns/callus	Loss of sensation
3.	Skin/nail infection	Tender / painful feet
4.	Neuropathy / loss of sensation	Drop foot
5.	Bunion	Low arch / flat foot
6.	Tender / painful feet	Swelling in feet / ankles
7.	Lesser toe deformity	Bunion
8.	Ingrown toe nails	Ingrown toe nails
9.	Arthritis	Corns/callus
10.	Plantarfasciitis	Toes curl up/under
11.	Vascular	Lesser toe deformity
12.	Chilblains, cold toes/feet	Fungal infections
13.	Gout	Problems with nail growth
14.	Ulceration	Arthritis
15.	Drop foot	Cramp
16.	Low arch / flat foot	Plantarfasciitis
17.	High arch foot	Problems due to differences in leg length/foot size/ shoe size

### Falls status

Responses differed when the sample was split according to fall status (see Table 4). In comparison to non-fallers a significantly greater proportion of fallers reported foot problems, which they stated influenced balance, led to changes in footwear habits and influenced the type of shoes they wear (all *p* < 0.01). Eighty-four fallers reported that they mostly wore slippers and walked barefoot indoors; of these 43 (51 %) received foot care support and 41 (49 %) did not. A greater proportion of those who reported falls would like more advice and choice when buying new shoes and a greater percentage of fallers than non-fallers highlighted fastening of shoes as important (both *p* < 0.01). Although a greater proportion of fallers (53 %) than non-fallers reported receiving foot care help (37 %), that does mean that 47 % of fallers report not receiving foot care support. Further investigation of the data revealed that, of those who report both falls and foot problems, 36 % reported that they had not received help with foot care.

**Table 4:** Survey answers for fallers and non-fallers relating to foot problems and footwear

		Fallers (*n* = 180)	Non-fallers (*n* = 183)	*P* Value
Months since diagnosis^a^	mean (SD)	75 (75.2)	44 (47.5)	<0.001
Do you receive foot care help from a health professional?^b^ (% Yes)		96 (53 %)	67 (37 %)	0.001
Have your footwear habits changed since diagnosis?^b^ (% Yes)		96 (53 %)	68 (37 %)	0.003
Foot problems?^b^	any problem	103 (57 %)	75 (41 %)	0.002
	influencing balance	61 (34 %)	39 (21 %)	0.018
	influencing shoes worn	69 (38 %)	40 (22 %)	0.001
Would like more choice when buying footwear?^b^ (% agree)		86 (48 %)	66 (36 %)	0.024
Would like more advice when buying footwear?^b^ (% agree)		84 (47 %)	50 (27 %)	<0.001
Factors influencing indoor footwear purchase^b^ (% ranking factor ‘important’)	Comfort	156 (87 %)	159 (87 %	0.810
	Style	37 (21 %)	28 (15 %)	0.556
	Fashion	7 (4 %)	10 (6 %)	0.464
	Secure fastening	102 (57 %)	77 (42 %)	0.008
	Ease of fastening	103 (57 %)	73 (40 %)	0.007
	Support	96 (53 %)	95 (52 %)	0.484
	Grip	97 (54 %)	105 (57 %)	0.966
	Fit	140 (78 %)	141 (77 %)	0.477
Factors influencing outdoor footwear purchase^b^ (% ranking factor ‘important’)	Comfort	164 (91 %)	160 (87 %)	0.139
	Style	51 (28 %)	31 (17 %)	0.124
	Fashion	20 (11 %)	16 (9 %)	0.591
	Secure fastening	20 (62 %)	104 (57 %)	0.434
	Ease of fastening	117 (65 %)	93 (51 %)	0.010
	Support	123 (68 %)	118 (65 %)	0.761
	Grip	122 (68 %)	122 (67 %)	0.896
	Fit	152 (84 %)	146 (80 %)	0.379
Functional Ambulation Category^b^	Nonfunctional,	4 (2 %)	8 (4 %)	0.001
	Dependent, Level II	15 (8 %)	10 (6 %)	
	Dependent Level I	24 (13 %)	14 (8 %)	
	Dependent Supervision	14 (8 %)	6 (3 %)	
	Independent, level surface	57 (32 %)	43 (24 %)	
	Independent, all surfaces	57 (32 %)	100 (55 %)	

Figures are number(%) unless stated otherwise

Answers shown in % yes unless otherwise indicated

^a^ Analysed using Mann–Whitney *U* test

^b^ Analysed using Pearson chi-square test comparing yes/no, agree/disagree or important/not important responses and comparison of proportion of people in the different categories in the Functional Ambulation Category

## Discussion

To our knowledge this is the first study that has explored choice of indoor and outdoor footwear in relation to self-reported foot problems and fall status in people with stroke (PwS) and Parkinson’s (PwP). Since diagnosis/onset of their condition nearly half of the survey respondents reported that their footwear habits had changed. Given the manifestations of Parkinson’s and stroke and their effect on balance and mobility [29, 30] these changes are not surprising but as footwear habits have not been previously explored in these populations we cannot compare our findings to other published studies.

Over half of our participants reported foot problems. This is similar to evidence for ‘older adults’ in which many report some form of foot problem [31, 32] and this is potentially associated with mobility loss [33] and falls [32, 34]. Our results suggest that those who reported foot problems have lived with their condition for a long time and potentially their problems may have developed through long term use of inappropriate footwear and lack of foot care support. A link between foot problems and falls in the general population is well reported in the literature [2, 3, 30, 33]. It is well known that PwS and PwP are at higher risk of falls than people among older adults [17–19]. Findings from this study add new information highlighting that foot problems are also common in this patient group, possibly increasing the risk of falls. When all participants combined over both condition groups were compared according to fall status, a higher proportion of fallers reported that foot problems impacted on their balance and choice of footwear, and a greater proportion had changed their footwear habits since diagnosis.

Foot problems reported by PwS and PwP in the present study largely correspond with previous observations [23–27, 35]. The impact of stroke related foot problems such as weakness, lack of sensation and pain are likely to be increased when wearing inappropriate shoes. This suggests that it may be possible to decrease the impact of these problems through the use of more supportive and appropriate footwear. To date, few studies have explored impact on different types of shoes on balance and walking ability in PwP but initial findings suggest improved balance performance in supportive and outdoor shoes [36, 37]. We could not find any literature relating to specific foot problems in PwP.

With respect to footwear, the most important factors in decisions for purchasing indoor and outdoor shoes were based on comfort and fit. Fallers placed a particular emphasis on ease and security of the fastening. Most respondents reported that they wear lace up walking shoes outdoors. In contrast, in their systematic review, Menant et al. [13] highlighted that many older adults wore inappropriately fitting shoes both inside and outside the home. Although ‘lace up walking shoes’ are deemed a ‘good’ footwear choice [10], our postal survey design did not permit a review of the condition or fit of the actual shoes worn by participants who reported this choice of footwear.

Footwear is known to influence falls in older adults [13, 14]. Wearing slippers and/or walking barefoot indoors, puts individuals at greater risk of falls [11, 12, 15]. In spite of the substantial advances in the fields of footwear design and falls research since the turn of the century [13, 14] our study showed that slippers were still the type of shoes most respondents wear indoors and that many believed that these were right for them. This finding might be explained at least partially on the fact that purchasing and wearing of potentially unsafe household shoes is based on long ingrained habits. Household shoes are often received as presents or purchased when they are readily available if they are easy to put on, comfortable, fashionable and inexpensive [38]. It is also possible that the lack of footcare support reported by many respondents is linked to limited access to information about healthy footwear options. Worryingly, a large number of fallers wore slippers or walked barefoot indoors. Forty-seven percent of fallers and 36 % of fallers with foot problems reported that they had not received any foot care support or advice. We do not know why this is the case for our study participants especially as multifaceted podiatry interventions can enhance falls prevention strategies in older people [12]. In addition, the NICE guidelines for older people recommend that older people who have reported a fall or are at risk of falling should be offered a multifactorial falls risk assessment and monitored for balance and gait deficits [39]. It is not known whether all fallers in our survey had received such an assessment but based on the lack of input many reported, the question arises whether the possible influence of feet and footwear on balance are currently being overlooked. The need for more specific advice was supported by our findings that a third of PwP, over 40 % of PwS and nearly 50 % of fallers reported a need for more advice and support in their decision making when purchasing shoes.

Our findings are similar to reports of podiatry intervention / foot care services for other long term conditions [40–43]. What was surprising is that there was no observable difference in footwear choices for those who did and did not receive foot care support in our study and that our findings suggest that the uptake of healthy footwear particularly in relation to indoor shoes choices is still low. What is not known is whether podiatrists and foot health clinicians take into account what the individual wears in different situations when giving footwear advice. For example, in relation to the underlying variations in foot manifestations due to the effects of Parkinsons or stroke and/or how PwP and PwS assimilate that advice. The healthcare professionals that PwP and PwS see most frequently in the UK are likely to be physiotherapists and nurses. There is evidence that whilst some physiotherapists are knowledgeable concerning fall risk assessment and prevention strategies, they are less likely to refer to other healthcare professionals to address fall risk [44]. In a recent review, Borland et al. [45] could not identify any UK or international standardised guidelines that advise nurses about appropriate and safe footwear for older people. They concluded that structured guidelines to direct nurse educators about what to teach student nurses concerning appropriate footwear for older people may work towards reducing falls [45].

Future research may also investigate optimal shoe design requirements and explore whether it is possible to improve foot health status in PwS and PwP. For example, Williams and Nester found differences in design requirements between patients with diabetes and patients with rheumatoid arthritis [46].

We acknowledge limitations of this study with regards to participant recruitment, the survey design and its reliance on self-report. Firstly, the response rate appears low (36 %). Although 1000 survey packs were distributed to health professionals and leads of Parkinson’s and stroke clubs we do not know how many survey packs actually reached individual patients. It is possible that amongst potential participants who received a survey pack the response rate was higher than our figures suggest. Secondly, participants were recruited from the wider Southampton area which is predominantly white and middle-class. The study findings may therefore be subject to selection bias with regards to geographical location, ethnicity and culture.

## Conclusion

Despite a decade of investigation and evidence that footwear is a modifiable factor that could be targeted in falls’ prevention strategies, our findings indicate that PwP and PwS are more likely to be wearing slippers whilst indoors. A high percentage of PwS and PwP reported both foot problems and falls impacting on footwear habits and choice of footwear, yet many do not receive foot care support. Our findings highlight an unmet need for foot health advice / foot care for PwS and PwP, a need for further research to explore the most appropriate mechanisms to provide access to foot care services and advice on safe and appropriate healthy footwear choices. Addressing these unmet needs may enhance effectiveness of existing falls prevention strategies.

## Additional files

Additional file 1: Questionnaire to survey people with stroke choice of indoor and outdoor footwear, foot problems and fall history. (DOC 575 kb) Additional file 2: Questionnaire to survey people with Parkinson’s choice of indoor and outdoor footwear, foot problems and fall history. (DOC 574 kb)

## Abbreviations

LREC: Local research ethics committee
NICE: National Institute for Health and Care Excellence
PwP: People with Parkinsons
PwS: People with stroke
R&D: Research and development
SHOES: People with Stroke and Parkinson’s: Home and Outdoor shoes’ project
