# Supplementary material for: A survey exploring self-reported indoor and outdoor footwear habits, foot problems and fall status in people with stroke and Parkinson’s
Source: J Foot Ankle Res. 2016 Sep 22;9:39. doi: 10.1186/s13047-016-0170-5 (PMC5034630; doi:10.1186/s13047-016-0170-5)
Supplement: Additional file 1: — Questionnaire to survey people with stroke choice of indoor and outdoor footwear, foot problems and fall history. (DOC 575 kb) [file 13047_2016_170_MOESM1_ESM.doc]

Questionnaire for people with Parkinson’s disease

|  | Please could you tell us:   1. Your age ………… b) Your gender (please tick)  Male  Female 2. How long ago you have been diagnosed with PD? …………Years………………Months. 3. Have you had any falls in the last 12 months? (please tick **one box**)   No  Yes: One fall  Yes: 2+ falls   1. Who lives with you? (Please read the list below and tick **one** box that best describes your living status)   No one I live on my own  Partner  Family/Friends  Warden controlled/Assisted living. Please describe…………………………………………….  Other Please describe…………………………………………………........................................   1. Do you need help from other people to walk? (Please tick **one** box that best describes your mobility). It does not matter if you use a walking aid to achieve this, we will ask about aids next.   I cannot walk or need help from 2+ people  I can walk with lots of help from 1 person  I can walk with a little help from 1 person  I can walk with supervision (no physical help)  I can walk independently on level ground  I can walk independently anywhere   1. Do you use walking aids?  No  Yes: Please describe…………………………………… 2. Do you feel that your feet get glued to the floor while walking, turning or starting to walk? (Please   tick **one** box that best describes your freezing episodes).  never about 1x month about 1x per week about 1x per day  always (whenever walking)   1. How far can you walk outdoors? (Please tick **one** box that best describes your outdoor walking)   I cannot walk outside I can only walk to the car/garden gate I can walk less than ¼ mile  I can walk further than ¼ mile but no further than 1 mile    I can walk more than 1 mile |
| --- | --- |
| **2.** | **Please could you tell us:**   1. Have you got any problems with your feet?  No  Yes: Please describe………………….   …………………………………………………………………………………………………………………..   1. Do you see any health professionals for your foot care?  No  Yes   Please describe……………………………………………………………………………………………………  **If you do not have any foot problems please skip to section 3**   1. Do your foot problems impact on your walking or your balance?  No  Yes   Please describe……………………………………………………………………………………….   1. Do your foot problems influence the type of shoes you wear?  No  Yes   Please describe………………………………………………………………………………………. |
| **3.** | **Please could you tell us:**   1. Which type of shoes do you wear **indoors**? (Please **circle** **all types** of shoes you wear **indoors**). 2. Now put a **X** in the box that best describes the type of shoes you currently **wear most often** **indoors**)?  |  |  |  |  |  | | --- | --- | --- | --- | --- | | **Wear most often** | **Wear most often** | **Wear most often** | **Wear most often** | **Wear most often** | | **Walking boot** | **Soft boot** |  |  |  | | **Wear most often** | **Wear most often** | **Wear most often** | **Wear most often** | **Wear most often** | |  |  |  |  | **NONE**  **Walk in socks or**  **Bare foot** | | **Wear most often** | **Wear most often** | **Wear most often** | **Wear most often** | **Wear most often** |     **Please could you tell us:**   1. Which type of shoes do you wear **outdoors**? (Please **circle** **all types** of shoes you wear **outdoors**). 2. Now put a **X** in the box that best describes the type of shoes you currently **wear most often outdoors**)?  |  |  |  |  |  | | --- | --- | --- | --- | --- | | **Wear most often** | **Wear most often** | **Wear most often** | **Wear most often** | **Wear most often** | | **Walking boot** | **Soft boot** |  |  |  | | **Wear most often** | **Wear most often** | **Wear most often** | **Wear most often** | **Wear most often** | |  |  |  |  | **Other Please state** | | **Wear most often** | **Wear most often** | **Wear most often** | **Wear most often** | **Wear most often** |  1. What do you like about the shoes you wear most often? Please describe……………………………   …………………………………………………………………………………………………………………  ………………………………………………………………………………………………………………...   1. Have you got any problems with the shoes you wear most often? Please describe………………….   ………………………………………………………………………………………………………………….  ………………………………………………………………………………………………………………… |
| **4.** | 1. **Please read the statements below and circle the numbers that match how you feel:**   (1 = not important 2 = not sure 3=important)   - When I buy new **indoor** shoes I make my decisions based on:  | - Comfort | 1 | 2 | 3 | | --- | --- | --- | --- | | - Style | 1 | 2 | 3 | | - Fashion | 1 | 2 | 3 | | - Secure fastening (Velcro, laces etc.) | 1 | 2 | 3 | | - Ease of fastening | 1 | 2 | 3 | | - Support | 1 | 2 | 3 | | - Good grip | 1 | 2 | 3 | | - Fit | 1 | 2 | 3 | | - Other: Please state…………………………………………. | 1 | 2 | 3 |  - When I buy new **outdoor** shoes I make my decisions based on:  | - Comfort | 1 | 2 | 3 | | --- | --- | --- | --- | | - Style | 1 | 2 | 3 | | - Fashion | 1 | 2 | 3 | | - Secure fastening (Velcro, laces etc.) | 1 | 2 | 3 | | - Ease of fastening | 1 | 2 | 3 | | - Support | 1 | 2 | 3 | | - Good grip | 1 | 2 | 3 | | - Fit | 1 | 2 | 3 | | - Other: Please state…………………………………………. | 1 | 2 | 3 |  1. **Please read the statements below and circle the numbers that match how you feel:**   (1 = disagree 2 = not sure 3=agree)   | - I would like more choice when buying new shoes | 1 | 2 | 3 | | --- | --- | --- | --- | | - I would like more advice about choosing the right shoes | 1 | 2 | 3 | | - The shoes I am currently wearing indoors are right for me | 1 | 2 | 3 | | - The shoes I am currently wearing outdoors are right for me | 1 | 2 | 3 |   **Please could you tell us:**   1. Have your foot wear habits changed since you have been diagnosed with PD?  No  Yes   Please describe……………………………………………………………………………………….  …………………………………………………………………………………………………………..   1. If there is anything else you wish to tell us please do so here……………………………………   ………………………………………………………………………………………………………….  ………………………………………………………………………………………………………….  ……………………………………………………………………………………………………………  **Thank you for completing this questionnaire.**  Please only complete **section 5** if you are happy for us to contact you about the other study components. Otherwise, skip this section and return the questionnaire to us in the Freepost envelope provided (no stamp needed). |
| **5.** | **Reply Slip**  I would like further information about the other study components and I am happy for the researcher to contact me.  I agree for you to contact me (Please initial the box)  Please use the following contact details to contact me: (Please Print)   | Name: | | | --- | --- | | Telephone Number: | | | Address: | | |  | | |  | | | Today’s date: | E-mail: |   **If you only want to be contacted for one of the other study components please tell us:**  I am only interested in the personal views interview:  I am only interested in the foot and balance assessment:  Please return the completed reply slip in the Freepost envelope (no stamp needed).  Thank you very much for your interest. |
